# The ERK MAPK pathway in mesenchymal glioblastoma: tumorigenesis, microenvironmental reprogramming, and the therapeutic promise of RAS(ON) multi-selective inhibition

**DOI:** 10.3389/fnmol.2026.1857885

**Published:** 2026-08-18

**Authors:** Uma M. Basole, Mary M. Fitzpatric, Joseph Donohoe, Shashank Obulasetti, William Snider, Emily Haugen, Damir Garcia, Ana Gonzalez-Manteiga, Mathieu Sertorio, Marc Oria

**Affiliations:** 1 Department of Radiation Oncology, University of Cincinnati College of Medicine, Cincinnati, OH, United States; 2 Cincinnati Children’s Hospital Medical Center, Cincinnati, OH, United States; 3 Xavier University, Cincinnati, OH, United States; 4 University of Cincinnati Cancer Center (UCCC) and Brain Tumor Center (BTC), University of Cincinnati, Cincinnati, OH, United States

**Keywords:** daraxonrasib, ERK-MAPK signaling, glioblastoma, mesenchymal glioblastoma, *NF1*, RAS(ON) inhibitors, targeted therapy, tumor microenvironment

## Abstract

Glioblastoma (GBM) remains one of the most lethal malignancies in adults, with a median survival of 15 months under the current standard of care. The lack of effective targeted therapies is a critical gap, particularly for the mesenchymal subtype of glioblastoma (MES-GBM), which accounts for up to 49% of GBM cases and carries the worst prognosis. Although direct RAS mutations are rare in GBM, mutations in upstream ERK MAPK pathway regulators such as loss-of-function of neurofibromin 1 (*NF1*) and gain-of-function mutations in epidermal growth factor receptor (*EGFR*) are highly prevalent in MES-GBM and drive constitutive pathway hyperactivation, chemoresistance, and aggressive tumor behavior. These mutations render the ERK MAPK pathway a compelling, yet underexplored, therapeutic target in GBM. RAS(ON) multi-selective inhibitors, which act upstream by blocking active RAS-GTP across multiple isoforms and mutations, represent a new therapeutic opportunity. Daraxonrasib (RMC-6236), a potent RAS(ON) multi-selective inhibitor currently in Phase III clinical trials for pancreatic cancer, has demonstrated blood-brain barrier (BBB) penetrance in non-GBM brain metastasis models, broad efficacy across RAS-driven cancers, and a favorable tolerability profile in clinical studies. As daraxonrasib targets active RAS regardless of mutation status, it is mechanistically suited for *NF1*-mutant GBM, in which RAS itself is wild-type but constitutively activated. Beyond direct tumor cell effects, hyperactive RAS signaling in GBM drives pro-tumoral reprogramming of microglia and tumor-associated macrophages (TAMs), creating an immunosuppressive microenvironment that further promotes MES-GBM transition and treatment resistance. RAS(ON) inhibition therefore holds potential to simultaneously suppress tumor proliferation and remodel the tumor microenvironment (TME) toward an anti-tumor state. In this review, we discuss the potential effect of daraxonrasib as an emerging targeted therapeutic candidate in MES-GBM, highlighting the need of further research and clinical evaluation to better determine its therapeutic efficacy dedicated GBM preclinical models.

## 1 Introduction

Gliomas are the most common malignant tumors of the central nervous system, with an estimated annual incidence of 6 cases per 100,000 individuals (Mesfin et al., 2024). They represent a heterogenous group of primary brain tumors, classified from grade 1 (least severe) to grade 4 (highly malignant) according to the World Health Organization classification system (Mesfin et al., 2024). Glioblastoma (GBM), a grade 4 glioma, represents the most clinically challenging and aggressive subtype and accounts for approximately half of all adult malignant brain tumors (Mesfin et al., 2024). Per the 2021 WHO criteria, a GBM is defined as an IDHwt astrocytic glioma with microvascular proliferation or necrosis, and at least one of the following molecular features: gain of whole chromosome 7 and loss of chromosome 10 (+7/−10), *TERT* promoter mutation, or *EGFR* amplification (Berger et al., 2022). At the molecular level, the Cancer Genome Atlas (TCGA) classifies GBM into three transcriptional subtypes based on gene expression profiles: proneural, classical, and mesenchymal. Each subtype is associated with distinct molecular alterations, disease progression, and prognosis (Wang et al., 2018).

The current standard of care is surgical resection if feasible, radiotherapy (RT), and treatment with temozolomide (TMZ), a DNA alkylating agent based on the Stupp protocol first devised in 2005 (Davis, 2016). This protocol increased median survival from 12.1 months (with RT alone) to 14.6 months (Stupp et al., 2005). However, the median survival after diagnosis has remained at approximately 15 months, with fewer than 5% of patients surviving more than 5 years post-diagnosis (Davis, 2016). GBM is highly resistant to treatment due in part to enhanced DNA repair mechanisms within the tumor, hypoxia-mediated radiation resistance, and persistence of tumor stem-like cells (Yalamarty et al., 2023). New avenues of treatment are urgently needed, particularly targeted therapies capable of addressing the molecular drivers of GBM’s aggressive mesenchymal subtype, such as RAS(ON) multi-selective inhibitors.

Targeted therapy, wherein inhibitors of proto-oncogene expression or protein function are used as chemotherapeutic agents, has yielded significant benefits in cancers such as non-small-cell lung carcinoma (NSCLC) (Jeon et al., 2025; Lee et al., 2018). However, pharmacologic targeted therapy remains underexplored in GBM. As of 2025, the only FDA-approved targeted therapy for high-grade gliomas is bevacizumab, a vascular endothelial growth factor (VEGF) inhibitor, and for recurrent GBM only (Fisher and Adamson, 2021). As of June 2026, bevacizumab is currently in clinical trials as a monotherapy for recurrent GBM administered through intra-arterial cerebral infusion (NCT01269853). In this review, we discuss the molecular rationale for targeting the upstream signaling of the ERK MAPK pathway in mesenchymal GBM (MES-GBM), the microenvironmental consequences of NF1 loss, challenges imposed by the blood-brain barrier, and the preclinical and clinical evidence supporting daraxonrasib, a RAS(ON) multi-selective inhibitor, as a candidate therapeutic warranting dedicated evaluation in GBM models.

## 2 Mesenchymal GBM and ERK MAPK pathway activation

Glioblastomas are classified into three transcriptional subtypes including classical, proneural, and mesenchymal, which reflects dynamic molecular states rather than fixed diagnostic entities (Wang et al., 2018). The mesenchymal subtype of glioblastoma (MES-GBM) is defined by a gene expression signature driven by master transcriptional regulators including STAT3 and C/EBPβ, upregulation of mesenchymal markers such as CD44 and YKL-40, high NF-κB pathway activity, and frequent loss or inactivation of NF1. These molecular features are associated with greater invasiveness, vascular remodeling, immune-cell infiltration, and resistance to therapy compared to the classical and proneural subtypes (Davis, 2016; Wang et al., 2018). MES-GBM carries a substantially worse prognosis than other GBM subtypes: median survival is approximately 11.5 months compared to 14.4 months for non-MES GBM (Marques et al., 2021; Wang et al., 2018). Mesenchymal glioblastomas (MES-GBM) exhibit a stem-cell-like phenotype, spread aggressively, and are particularly difficult to treat (Vadla et al., 2023; Yalamarty et al., 2023). Approximately 30%–49% of GBM patient samples have been classified as mesenchymal (Behnan et al., 2019).

NF1 is more frequently mutated in MES-GBM than in other GBM subtypes: NF1 loss-of-function mutations are observed in 32% of MES-GBM samples versus 15% of non-MES-GBM samples (Marques et al., 2021). EGFR amplification through gain-of-function mutation is also observed in around 60% of primary GBMs and is mutated in 29 percent of MES-GBM (Oprita et al., 2021; Xu et al., 2017). NF1 and EGFR are both upstream regulators of the ERK MAPK pathway, though through distinct mechanisms: NF1 loss-of-function leads to impaired RAS-GTP hydrolysis and constitutive RAS activation, while EGFR acts through amplification or gain-of-function mutation that drives receptor-level pathway hyperactivation.

Therapeutic targeting of downstream members of the ERK MAPK pathway has already demonstrated clinical relevance in glioma. MEK1/2, also known as MAPK, is a serine-threonine kinase within the canonical RAS-RAF-MEP-ERK cascade (Molina and Adjei, 2006). The MEK inhibitor (MEKi) trametinib in combination with dabrafenib has been FDA-approved as a therapeutic agent, in low-grade pediatric glioma with a BRAF V600E mutation, establishing MAPK pathway as clinical strategy in defined gliomas (Habibi et al., 2024). Furthermore, Trametinib has also demonstrated antitumor effects in a preclinical murine model of MES-GBM (Levy et al., 2025). These findings provide the rationale for evaluating therapeutic approaches targeting the ERK MAPK pathway, including RAS-directed inhibitors (RASi). Targeting the RAS protein, which affects all downstream members of the ERK MAPK pathway, and therefore pathways regulated by the ERK MAPK pathway could be highly effective for MES-GBM treatment and warrants further study.

## 3 NF1 loss and myeloid remodeling in GBM

The glioblastoma tumor microenvironment (TME) contains a prominent and heterogenous myeloid compartment that can contain up to 50% of GBM tumor cellularity (Zhang et al., 2021) This compartment includes brain-resident microglia, bone marrow-derived monocyte-recruited macrophages, and border-associated macrophages at perivascular and meningeal niches (Sharma et al., 2023). While each population plays distinct roles, they converge to regulate tumor invasion, angiogenesis, immune suppression, and therapeutic resistance. In this context, the MES-GBM myeloid compartment is relevant because it directly connects NF1 loss in the tumor and RAS/MAPK hyperactivation with the promotion of myeloid recruitment and reprogram within the TME. The inferior outcome of MES-GBM is attributed to its extensive vascularization, rapid growth, and high levels of microglia, macrophage, and lymphocyte infiltration (Vadla et al., 2023; Zhang et al., 2021).

### 3.1 Microglia

Microglia are the resident immune cells of the central nervous system, playing a crucial role in maintaining homeostasis (Tripathy et al., 2024). In GBM, microglia are primarily enriched at the invasive tumor margin, where they interact with migrating tumor cells and extracellular matrix (ECM) components. Infiltrating bone marrow-derived macrophages predominate in the hypoxic and necrotic tumor core (Tripathy et al., 2024). Within GBM, microglia adopt pro-tumoral phenotypes that support tumor growth, invasion, and angiogenesis, releasing cytokines and growth factors that suppress immune responses and maintain mesenchymal-like tumor states (Tripathy et al., 2024). Microglia associated with GBM play a critical role in treatment resistance and increase radioresistance by secreting cytokines such as IL-6, and upregulating drug efflux pumps to combat chemotherapy drugs (Tripathy et al., 2024).

**FIGURE 1:**
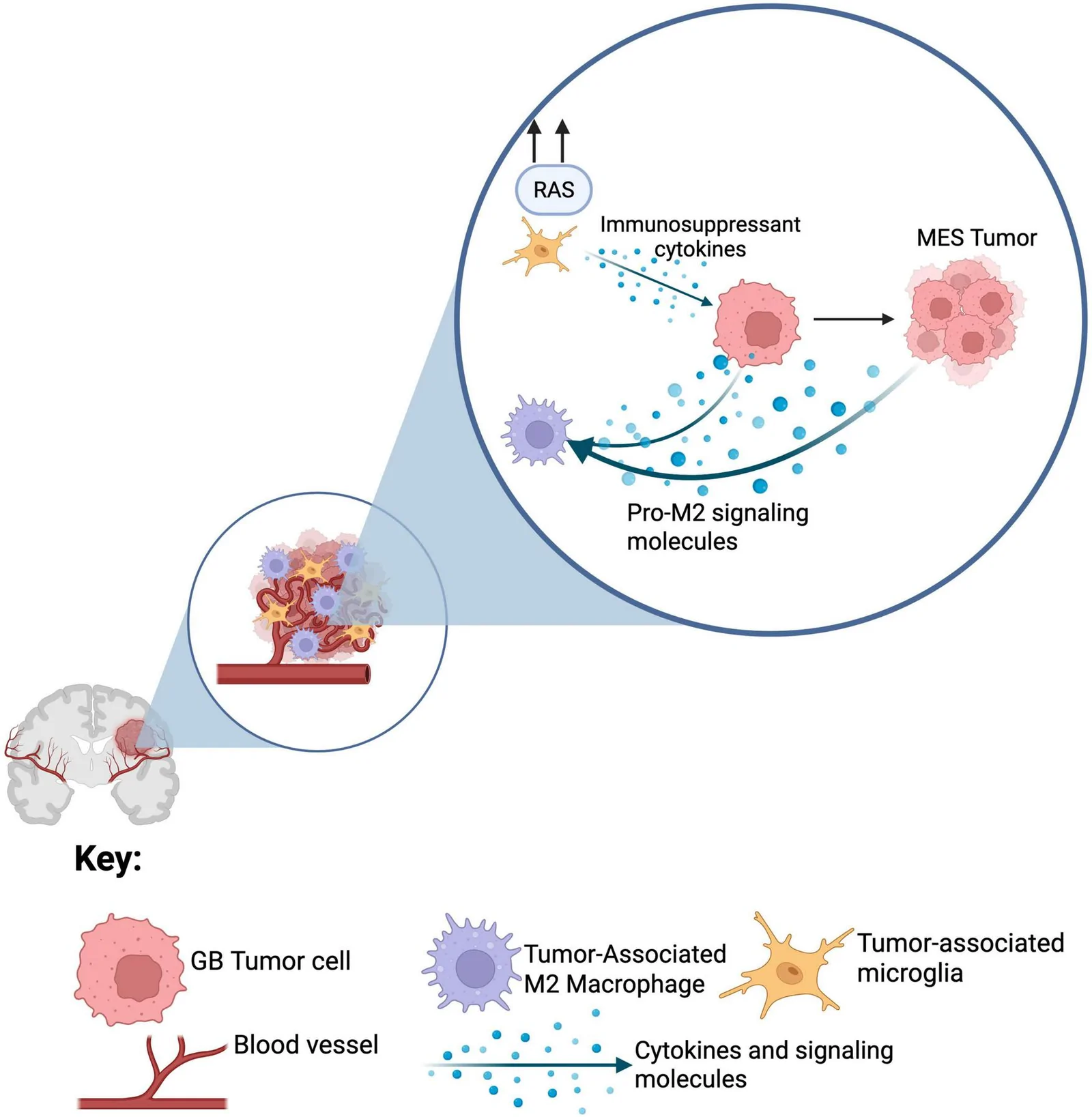
The glioblastoma (GBM) microenvironment. The tumor microenvironment includes GBM tumor cells, tumor-associated microglia, M2 macrophages, and blood vessels. Microglia secrete growth factors and cytokines that drive tumor transition into the mesenchymal GBM phenotype. Anti-inflammatory M2 macrophages aid angiogenesis and tumor migration. The pro-tumor activity of the microglia is RAS-mediated. The tumor secretes cytokines and signaling molecules that cause macrophages to polarize into the pro-tumor M2 phenotype. Created in BioRender. Basole, U. (2026) https://BioRender.com/0xu9niy.

Therapeutic stress further reshapes microglial function. Following radiotherapy (RT), hypoxia-inducible factor 1-alpha (HIF-1α)-mediated signaling and GBM-derived exosomes further polarize microglia toward tumor-supportive phenotypes like that of M2 macrophages, contributing to tumor therapy resistance (Gabrusiewicz et al., 2018; Yu et al., 2020). These states of microglia are associated with reduced expression of lineage-specific markers such as TMEM119, P2RY12, and FCRLS within the TME, acquiring instead a disease-associated transcriptional profile characterized by upregulation of phagocytic and inflammatory genes that predominantly support tumor progression (Bowman et al., 2016). This homeostatic marker loss is accompanied by upregulation of genes associated with an activated, tumor-supportive state, including *SPP1*, *CD44*, and *LGALS3*, reflecting a shift away from surveillance functions toward microenvironmental remodeling that favors tumor growth (Wang et al., 2017). Importantly, the GBM-associated microglial landscape is not a simple binary between pro- and anti-tumoral states, but rather a continuum of functional phenotypes; recent evidence has identified distinct microglial subpopulations with anti-tumoral activity within GBM, underscoring the complexity of this compartment and the need for further characterization of specific pro- and anti-tumoral microglial signatures as potential immunomodulatory strategies (Ma et al., 2022; Wang S. et al., 2024). NF1 loss in GBM promotes microglial proliferation, pro-inflammatory cytokine production, and phenotypic change toward tumor-supportive states (Mirzaei et al., 2026; Ochocka et al., 2021; Wang et al., 2017). In the post-radiotherapy setting, this could contribute to radiation-induced brain injury in patients undergoing treatment for GBM, manifesting as cognitive decline, white matter damage, and potentially accelerated tumor recurrence by creating a pro-tumorigenic microenvironmental state (Ochocka et al., 2021). This suggests that GBM-driven microglial reprogramming co-opts a broader microglial activation axis (Ma et al., 2022). The effects of ERK MAPK activation on the GBM microenvironment are illustrated in Figure 1.

### 3.2 Tumor-associated macrophages

Tumor-associated macrophages (TAMs) in GBM comprise two distinct populations: brain-resident microglia-derived macrophages, which predominate at the tumor margin, and peripheral monocyte-derived macrophages recruited from the bloodstream, which accumulate in the hypoxic tumor core (Solomou et al., 2024). While the M1/M2 polarization framework is used for simplicity, current evidence indicates that TAMs exist along a spectrum of functional states rather than two discrete phenotypes. In GBM, TAMs are predominantly skewed toward immunosuppressive, pro-tumorigenic states that support tumor growth, invasion, and angiogenesis (Rao et al., 2022; Sharma et al., 2023; Solomou et al., 2024). Tumor-derived signals such as growth factors and cytokines, recruit TAMs to the tumor site. NF1 loss-of-function in GBM provides a mechanistic link between upregulated RAS/MAPK signaling, and myeloid remodeling (Rao et al., 2022; Solomou et al., 2024). In MES-GBM tumor cells upregulates the expression of chemokines, including CX3CL1 (C-X3-C motif chemokine ligand 1), CCL2 (C-C motif chemokine ligand 2), MIC-1 (macrophage inhibitory cytokine-1), and CSF1 (colony-stimulating factor 1). CX3CL1 and CCL2 act as monocyte chemoattractants that recruit circulating monocytes to the tumor site; CSF1 promotes their differentiation and survival as M2-like TAMs; and MIC-1 suppresses pro-inflammatory macrophage activation. Together, these factors create a feedforward loop that sustains an immunosuppressive, pro-tumorigenic myeloid TME (Andersen et al., 2021).

Hypoxia and tumor-derived exosomes further reinforce tumor-supportive TAM states. These TAMs populations suppress cytotoxic T-cell and natural killer (NK) cell activity, facilitating immune evasion (Rao et al., 2022). Transforming growth factor-β (TGF-β), secreted by both GBM tumor cells and TAMs themselves, amplifies immunosuppression, angiogenesis, and extracellular matrix (ECM) remodeling (Andersen et al., 2021; Rao et al., 2022). TAM-derived metalloproteases (MMP) (Rao et al., 2022), including MMP9 and MMP14, enzymes that degrades structural ECM components further contributes to tumor invasion and migration (Rao et al., 2022). In addition, TAMs also secrete growth factors such as VEGF and interact with oncogenic pathways such as EGFR and PI3k/Akt/mTOR that drive tumor progression (Andersen et al., 2021).

Given the role of NF1 loss in promoting ERK MAPK pathway activation and myeloid remodeling, upstream RAS inhibition (RASi) may represent a rational strategy for both tumor and TME in MES-GBM. In the microenvironment of MES-GBM, the changes driven by NF1 loss could possibly be mitigated by treatment with RASi drugs. Daraxonrasib, a RAS(ON) multi-selective inhibitor, has been reported to remodel the tumor microenvironment and promote antitumor immunity in a preclinical mouse model of RAS-mutant melanoma (Anastacio Da Costa Carvalho et al., 2026). Based on this evidence from RAS-mutant melanoma, daraxonrasib could hypothetically exert similar TME-remodeling effects in NF1-mutant MES-GBM; however, this remains entirely speculative in the absence of GBM-specific data. Future research using dedicated orthotopic MES-GBM models is needed to further characterize the effects of RAS(ON) inhibition on both GBM progression and the tumor microenvironment.

## 4 The ERK MAPK pathway and upstream regulation in GBM

The ERK MAPK pathway, also referred to as RAS-RAF-MEK-ERK, is a well-characterized signal transduction pathway controlling proliferation, survival, differentiation, migration, and angiogenesis (Molina and Adjei, 2006). In humans, RAS signaling is mediated by four protein isoforms: HRAS, NRAS, KRAS 4A, and KRAS 4B (Song et al., 2023). Pathway activation begins when a signal molecule binds to a protein tyrosine kinase receptor (RTKs), causing RAS to switch from its inactive GDP-bound state (RAS-GDP) to its active GTP-bound state (RAS-GTP) (Song et al., 2023). Active RAS then engages RAF kinases, which phosphorylate and activate MEK 1/2, which in turn activates and subsequently phosphorylates ERK 1 and ERK/2 (Song et al., 2023) enabling ERK-dependent regulation transcription factors such ELK1, AP-1, MYC and ETS family (Marei et al., 2021; Song et al., 2023).

The ERK MAPK pathway governs cellular processes central to tumor initiation and progression; aberrant activation can be potentially oncogenic (Molina and Adjei, 2006; Prior et al., 2020). Mutations in HRAS, NRAS, or KRAS are common in several human cancers, found in 72%–90% of pancreatic cancers and 15%–50% of lung cancers (Molina and Adjei, 2006). Overall, 20%–30% of all human cancers harbor a mutation in a RAS gene isoform (Prior et al., 2020). Mutations in genetic regulators of the ERK MAPK pathway, such as NF1 and EGFR, can produce a RAS-upregulated phenotype even without a mutation in RAS itself (Philpott et al., 2017).

**FIGURE 2:**
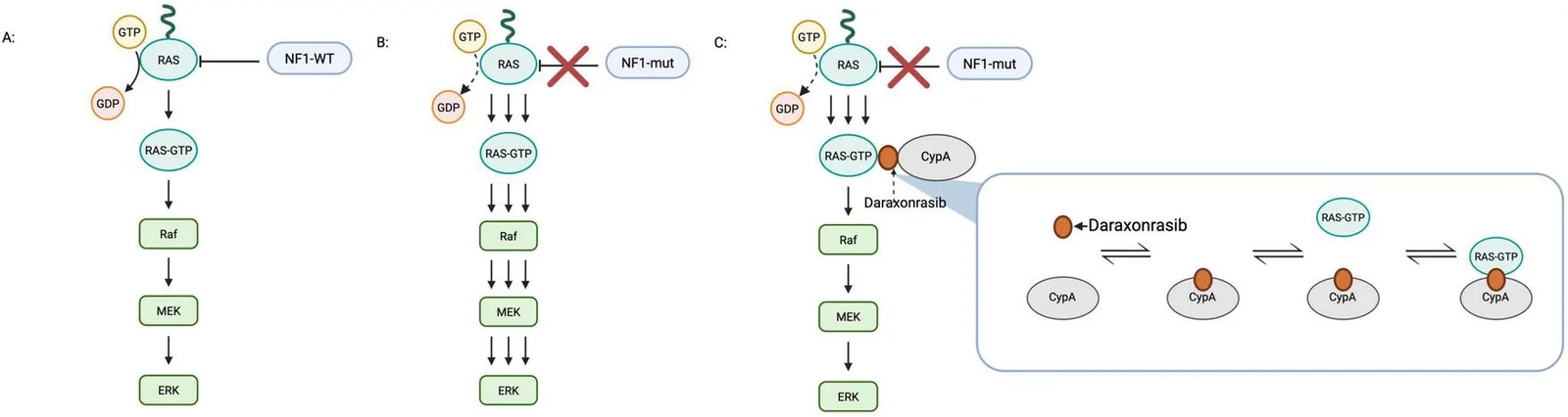
Effects of neurofibromin 1 (NF1) on RAS and ERK MAPK pathway. **(A)** The normal NF1 protein acts as a negative regulator of RAS and the ERK MAPK pathway, preventing overactivation and cell overproliferation. **(B)** In NF1-mutant glioblastoma, a loss-of-function mutation renders NF1 unable to regulate RAS, leading to RAS overactivation, cell overproliferation, and tumorigenesis. **(C)** Daraxonrasib, by forming a tricomplex with CypA and RAS-GTP (zoomed in), inhibits expression of genes further along the ERK MAPK pathway and reduces cell overproliferation. Created in BioRender. Basole, U. (2026) https://BioRender.com/xfmsr7y.

NF1 negatively regulates the ERK MAPK pathway by stimulating GTPase-activating proteins that convert active RAS-GTP to inactive RAS-GDP (Anastasaki et al., 2022). Loss-of-function mutations in NF1 have been observed in many RAS-associated tumors, which also respond to MEK inhibitors, consistent with the role of MEK as a downstream ERK MAPK pathway member (Anastasaki et al., 2022). The effects of NF1 loss-of-function on ERK MAPK pathway activity are illustrated in Figure 2.

Epidermal growth factor receptor is another upstream regulator of the ERK MAPK pathway, with oncogenic gain-of-function mutations distinct from the loss-of-function mechanism seen in NF1 (Xu et al., 2017). EGFR amplification or activating mutation drives signaling at the receptor level, promoting downstream RAS–RAF–MEK–ERK and PI3K–AKT–mTOR pathway activation (Oprita et al., 2021). In other tumor types, including RAS-mutant colorectal cancer (CRC), concurrent EGFR inhibition and RAS inhibition have shown superior treatment outcomes compared to either treatment alone in both mouse models and clinical trials supporting the concept that vertical pathway suppression may be required to overcome adaptive feedback and pathway reactivation (Krauß et al., 2025; Mizukami et al., 2019). These observations provide a mechanistic rationale for evaluating upstream ERK/MAPK pathway targeting strategies in molecularly selected GBM contexts, particularly NF1-deficient or MAPK-activated mesenchymal GBM.

## 5 Blood-brain and blood-tumor barriers and GBM therapy

One of the most significant obstacles to GBM treatment is the blood-brain barrier (BBB) (Ahmed et al., 2023), a specialized neurovascular interface that restricts the entry of circulating molecules into the central nervous system, preserving neural homeostasis (Ahmed et al., 2023). The BBB is composed of endothelial cells connected by tight junctions, supported by pericytes, astrocyte endfeet, basement membrane components and associated immune and stromal cells. Together, these cellular and structural elements regulate molecular transport, limit passive diffusion, and actively exclude many therapeutic agents through efflux transporters (Ahmed et al., 2023).

In GBM, the BBB is regionally disrupted as the tumor progresses and induces aberrant neovascularization, giving rise to a heterogeneous blood-tumor barrier (BTB) (Sarkaria et al., 2018). The BBB, prevents some therapeutic agents from reaching the central nervous system, severely limiting treatment options (Ahmed et al., 2023). In GBM the abnormal vasculature is disorganized with variably permeable, allowing partial drug delivery in some tumor regions with more glioma-associated neovascularization (Digiovanni et al., 2024; Sarkaria et al., 2018). Compared to other subtypes of GBM, MES-GBM is highly vascularized, which contributes to its worse prognosis (Vadla et al., 2023; Zhang et al., 2021). The resulting disorganized and irregular BTB allows partial chemotherapy penetrance compared to other brain cancers (Sarkaria et al., 2018). However, the BBB remains clinically relevant: due to the irregularities of glioma-associated neovascularization, some tumor regions retain BBB protection while others are exposed, leading to incomplete drug penetrance, tumor persistence, and poor prognosis (Ahmed et al., 2023; Sarkaria et al., 2018). Accordingly, preclinical evaluation of candidate GBM therapies requires orthotopic models capable of recapitulating tumor growth within the brain microenvironment and enabling pharmacokinetic and pharmacodynamic assessment of CNS exposure (Iturrioz-Rodríguez et al., 2024; Yaghoubi et al., 2026).

Any candidate targeted therapy for GBM must therefore demonstrate the capacity to cross the BBB and meaningful intratumor exposure. Temozolomide (TMZ), the current standard of care for GBM, is a small and lipophilic molecule that can cross the BBB (Ortiz et al., 2021). However, its clinical efficacy is limited by multiple resistance mechanisms, including tumor drug resistance that is supported by the TME, which is altered to be immune suppressive and promoting survival by NF1 loss and subsequent ERK MAP overactivation (Guo et al., 2019). If a promising agent cannot cross the BBB and BTB and demonstrates insufficient brain penetrance, then delivery enhancing approaches may be required to improve therapeutic exposure.

**FIGURE 3:**
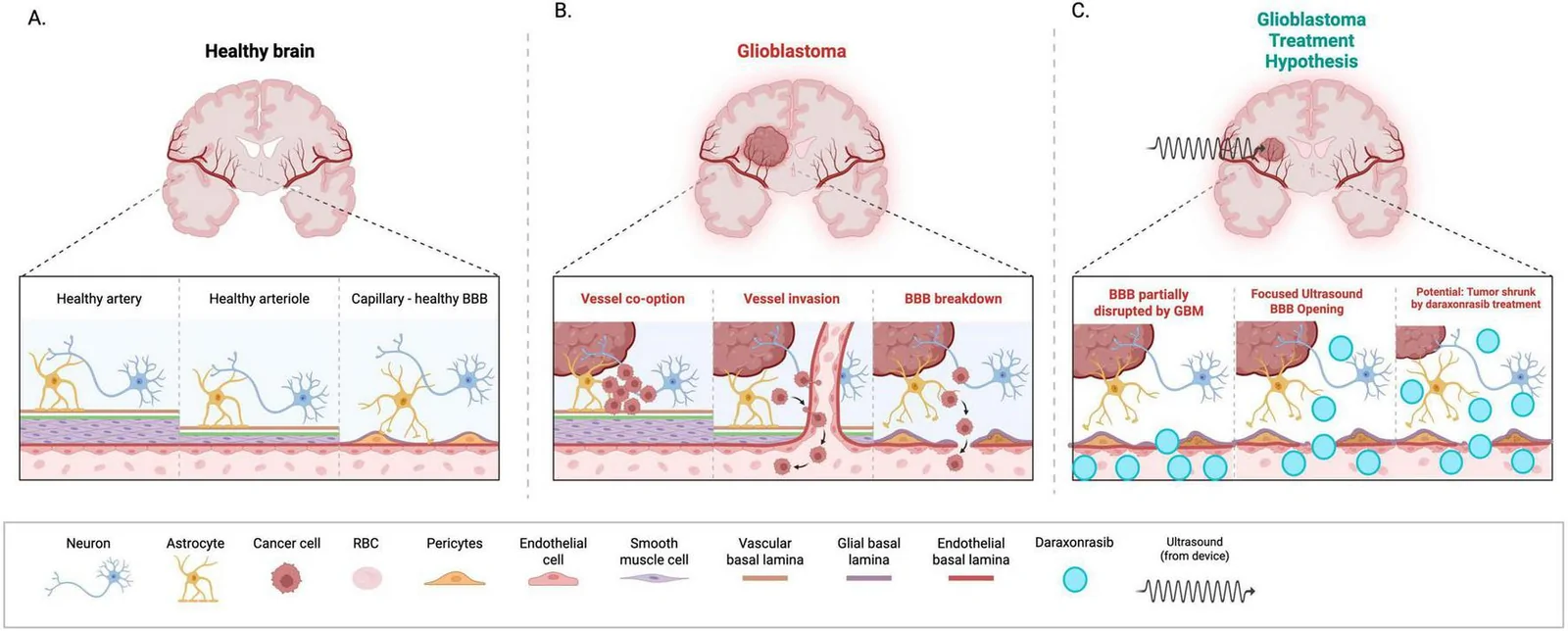
The blood-brain barrier in glioblastoma (GBM) and hypothetical use of blood-brain barrier disruption in treatment. **(A)** The normal, healthy blood-brain barrier (BBB) protects protect the brain from foreign molecules and maintain neural homeostasis. **(B)** In glioblastoma, the tumor disrupts the BBB in irregular ways, through glioma-associated neovascularization. The blood-brain barrier is only partially disrupted in GBM. **(C)** The partial disruption of the BBB in GBM does not allow full penetrance of drugs, even those that can theoretically pass through the BBB, in this case daraxonrasib. Controlled BBB disruption, such as by focused ultrasound as depicted, could hypothetically allow full treatment penetrance. Created using a BioRender template. Created in BioRender. Basole, U. (2026) https://BioRender.com/74i04zt.

Many novel approaches to circumvent the BBB have been developed to bypass or transiently disrupt the BBB, including nanoparticles, focused ultrasound, intranasal administration, deep brain stimulation, device implants, and direct intracerebral injection (Terstappen et al., 2021; Wu et al., 2023). As of 2024, intratumoral injections, intracerebral injections, nanoparticle delivery, are all in clinical trials as drug delivery mechanisms and potential treatments (Duan et al., 2024). From 2014 to 2018, the safety of ultrasound devices, specifically the Sonocloud device, in transiently opening the BBB was tested in Phase 1 and 2 clinical trials (Idbaih et al., 2019) (NCT02253212), and then another Phase 1 and 2 trials with the Sonocloud-9 device (Carpentier et al., 2024) (NCT03744026). In both trials, the Sonocloud and Sonocloud-9 devices were well-tolerated, and transient BBB disruption from the Sonocloud-9 device was correlated with longer progression-free survival in patients, suggesting improved drug delivery (Carpentier et al., 2024; Idbaih et al., 2019). In 2024, a Phase 3 clinical trial to test the efficacy of the Sonocloud-9 device in comparison with the current standard of care (NCT05902169) begun, and it is estimated to end in 2028. The possible use of focused ultrasound in improving drug penetrance in GBM is illustrated in Figure 3.

## 6 Potential of daraxonrasib/RMC-6236 in GBM treatment

RAS proteins were long considered difficult therapeutic targets because of their high affinity for GDP and GTP, rapid nucleotide cycling and their structural features (Moore et al., 2020). RAS has a picomolar affinity for GTP/GDP, leaving no accessible allosteric binding pocket, its smooth surface lacking the deep hydrophobic grooves typical of targetable proteins (Han et al., 2017). Its rapid GTP cycling kinetics prevent stable inhibitor binding, leading to the use of downstream inhibitors, targeting MEK and ERK, as therapeutic strategies for RAS-mutant cancers (Han et al., 2017; Lo, 2010; Moore et al., 2020). In 2021, the KRAS G12C inhibitor sotorasib received FDA approval for NSCLC treatment (Punekar et al., 2022), followed by adagrasib in 2022 (Punekar et al., 2022). However, resistance to both agents has emerged in NSCLC and other RAS-mutant cancers, and neither drug targets HRAS, NRAS, or non-G12C KRAS mutations (Nokin et al., 2024). Mechanistically, sotorasib and adagrasib target RAS-GDP (“off” RAS) by binding the cysteine substitution at the G12C position (Punekar et al., 2022).

This distinction is particularly relevant in GBM since RAS itself is rarely mutated in GBM (Lo, 2010), mutation-specific RASi drugs offer limited utility. In contrast, NF1 loss-of-function, which is commonly enriched in MES-GBM (Marques et al., 2021), promotes sustained constitutive RAS activation regardless of RAS mutation status. RAS-GTP inhibitors, specifically RAS(ON) multi-selective inhibitors, have been proven to act on wild-type RAS as well as mutant forms (Holderfield et al., 2024). This biology provides a rationale for investigating inhibitors that target active RAS-GTP rather than the mutation specific, resenting a new opportunity for GBM treatment.

### 6.1 Daraxonrasib (RMC-6236) mechanism of action

Daraxonrasib, also known as RMC-6236, is a potent, orally bioavailable RAS(ON) multi-selective inhibitor (Cregg et al., 2025). It was granted FDA orphan drug status in October 2025 for use in KRAS G12-mutant pancreatic ductal adenocarcinoma (PDAC) (Lee et al., 2022) and is currently in Phase III clinical trials for metastatic PDAC (NCT06625320). Mechanistically, daraxonrasib forms a tri-complex with RAS-GTP and Cyclophilin A (CypA), blocking RAF recruitment by steric occlusion and thus inhibiting ERK MAPK pathway propagation (Cregg et al., 2025). The mechanism of action of daraxonrasib is depicted in Figure 2 above. In mouse models, RAS-mutant lung tumors were significantly reduced in size upon daraxonrasib administration (Jiang et al., 2024). Importantly, daraxonrasib has also been observed to penetrate the BBB in a murine study, however, this was conducted in a brain metastasis model rather than a dedicated GBM model, and pharmacokinetic analysis was limited (Jiang et al., 2024; Perurena and Cichowski, 2024). To determine whether daraxonrasib can pass through the BBB in MES-GBM without treatments to circumvent it, it needs to be analyzed in a dedicated GBM, ideally NF1 mutated or MES-GBM model.

In clinical studies across RAS-mutant cancers (PDAC, NSCLC, CRC), daraxonrasib has been generally well-tolerated even at high doses, with most treatment-related adverse events (TRAEs) consisting of manageable rashes (Spira et al., 2023). Severe TRAEs were rare and infrequently required dose reduction (Spira et al., 2023).

### 6.2 Daraxonrasib (RMC-6236) as a candidate strategy for GBM treatment

Because RAS mutations are rare in GBM (Lo, 2010), direct RAS inhibition has not been considered a primary therapeutic approach for this tumor type. However, *NF1* loss-of-function sensitizes GBM tumors to MEK inhibitor drugs, as MEK is a downstream ERK MAPK pathway member regulated by NF1 (Chang et al., 2024). Several targeted therapies for ERK MAPK pathway members have been investigated in GBM, as summarized in Table 1.

**TABLE 1:** Drugs tested in glioblastoma, in mice and humans.

Compound	Mechanism of action	Status for use in GBM treatment	Blood-brain barrier penetrance	Synergy with other GBM drugs?
Temozolomide	DNA alkylating agent (Davis, 2016)	Primary chemotherapy in GBM (Davis, 2016; Fisher and Adamson, 2021)	Yes, but still relatively limited and can be improved (de Gooijer et al., 2018; Kumari et al., 2017)	Conflicting data about TMZ and bevacizumab combination therapy (Gerstner et al., 2020; van den Bent et al., 2018; Wei et al., 2024). Possible synergy with other drugs such as daraxonrasib, further testing is needed.
Bevacizumab	VEGF inhibitor (Fisher and Adamson, 2021; Ghiaseddin and Peters, 2015)	Currently used only in recurrent GBM (Fisher and Adamson, 2021); clinical trials as monotherapy for recurrent GBM ongoing: Phase 1 and 2 to end 2026–2027 (NCT01269853)	Does not penetrate intact BBB but can cross degraded BBB as present in GBM (Minichmayr et al., 2024)	Conflicting data about TMZ and bevacizumab combination therapy (Gerstner et al., 2020; van den Bent et al., 2018; Wei et al., 2024). Possible synergy with other drugs such as daraxonrasib, further testing is needed.
Fluvastatin	Prenylation inhibitor of RAS signaling (Long et al., 2022)	Tested only in mouse models; showed tumor inhibitory effects and overcame chemoresistance (Long et al., 2022)	Tested in mouse xenograft GBM models (Long et al., 2022)	Further testing is needed with fluvastatin and other drugs. Fluvastatin and TMZ have been observed to have synergy in one paper, more testing is needed (Long et al., 2022)
Erlotinib	EGFR inhibitor (van den Bent et al., 2009)	Completed Phase II trial (NCT00445588); insufficient as monotherapy (van den Bent et al., 2009)	BBB penetrance is poor despite small molecule size, will likely require BBB-circumventing devices and/or adjuvant therapies in further testing (Liang et al., 2023)	Possibly would synergize with daraxonrasib, testing needed
Trametinib	MEK inhibitor (Moore et al., 2020)	FDA-approved (with dabrafenib) in low-grade glioma (Habibi et al., 2024); no clinical trials for GBM despite efficacy in murine models (Levy et al., 2025)	BBB penetrance is poor without adjuvant drugs (Levy et al., 2025).	MEK is part of ERK MAPK pathway (Molina and Adjei, 2006). Combination therapy of trametinib and daraxonrasib would likely be redundant, but further testing is needed. Trametinib and dabrafenib are used in combination in low-grade glioma (Habibi et al., 2024)
Dabrafenib	BRAF kinase inhibitor (Habibi et al., 2024)	FDA-approved (with trametinib) in low-grade glioma (Habibi et al., 2024). No clinical trials for GBM despite efficacy in murine models (Levy et al., 2025).	Dabrafenib has higher BBB penetrance than other BRAFi but still low (Habibi et al., 2024). Synergistic with trametinib, possibly other drugs, further testing is needed.	Possibly would synergize with daraxonrasib, as daraxonrasib is RAS(ON) multi-selective inhibitor (Cregg et al., 2025). Further testing is needed.

Daraxonrasib is a RAS(ON) multi-selective inhibitor and should therefore function in cancers such as GBM that are generally not RAS-mutant, because it targets the active RAS-GTP state regardless of mutation status (Cregg et al., 2025). As daraxonrasib is a RAS(ON) multi-selective inhibitor, it may also reduce radiation-induced brain injury and microglia hyperactivation caused by ERK MAPK pathway activation (Wang Y. et al., 2024). Other drugs with off-target effects on RAS signaling, such as fluvastatin, have been tested in GBM and found to overcome chemoresistance (Long et al., 2022; Xu et al., 2024). This further supports the premise that modulation of the ERK MAPK axis can produce clinically relevant antitumor effects in GBM.

The molecule’s larger macrocyclic size may limit passive BBB diffusion, making co-administration with BBB-opening strategies such as focused ultrasound potentially necessary (Cregg et al., 2025). Therefore, daraxonrasib must be tested in a dedicated GBM model, before a definitive statement on its BBB penetrance can be made. PK/PD validation in orthotopic MES-GBM models should specifically assess brain-to-plasma drug ratio, tumor tissue concentrations at both the enhancing core and infiltrative margin, target engagement (reduction in RAS-GTP and pERK levels), and correlation with tumor growth inhibition, parameters that were not evaluated in the brain metastasis models where BBB penetrance was previously observed. An important and unresolved question is the optimal sequencing of daraxonrasib with radiotherapy: whether administration prior to RT could prime the TME toward an anti-tumoral state, whether concurrent use could sensitize tumor cells via ERK MAPK suppression, or whether post-RT delivery could counteract radiation-induced microglial reprogramming. These questions should be addressed in orthotopic GBM models before clinical translation.

An additional layer of complexity in MES-GBM treatment is the functional interplay between the ERK MAPK pathway and the PI3K/AKT/mTOR signaling axis. In GBM, upstream alterations such as EGFR amplification and PTEN loss, both prevalent in the mesenchymal transcriptional subtype, drive concurrent activation of both pathways, creating a network of redundant pro-survival signals (Shen et al., 2024). This crosstalk has direct therapeutic implications and inhibition of RAS or MEK can paradoxically relieve negative feedback on PI3K/AKT signaling, enabling compensatory AKT activation that sustains tumor cell survival and undermines monotherapy efficacy (Albert et al., 2009). Conversely, mTORC1 inhibition has been shown to activate ERK through IRS1-mediated feedback, further illustrating the bidirectionality of this crosstalk (Carracedo et al., 2008; Schreck et al., 2020). For daraxonrasib, which suppresses the ERK MAPK axis upstream at RAS-GTP, this compensatory PI3K/AKT rebound represents a plausible resistance mechanism that warrants prospective investigation. Combination strategies pairing daraxonrasib with PI3K, AKT, or mTOR inhibitors may therefore be necessary to achieve durable responses in MES-GBM and should be prioritized in future preclinical studies.

Another important factor to consider in GBM treatment is treatment resistance. In RAS-mutant cancers that are treated with RAS inhibitor monotherapy, resistance develops in the tumor (Ebright et al., 2025). While GBM are usually not RAS-mutant tumors, clinical testing is needed to determine whether they will follow similar resistance patterns as RAS-mutant tumors. Daraxonrasib treatment in MES-GBM, therefore, should be studied both as a monotherapy and as part of a combination with other drugs. This research would be useful to determine if daraxonrasib is more effective in combination with other treatments. If MES-GBM follows the same treatment resistance development patterns as other RAS-mutant tumors, then research into combination therapy would help mitigate that. Possible candidates include the EGFR inhibitor erlotinib, the BRAF kinase inhibitor dabrafenib, and other drugs in Table 1. Combinations of targeted therapy will need to be tested in vitro and in vivo to determine which treatments are most effective against the cancer, cause the least resistance in the tumor, and have the least side effects.

## 7 Conclusion

Mesenchymal glioblastomas remains a highly aggressive and lethal brain cancer for which treatment advances have been stagnant for two decades, and new therapeutic avenues must be urgently pursued. Its poor prognosis reflects both tumor intrinsic mechanisms and a complex microenvironment characterized by hypoxia, vascular remodeling, myeloid infiltration, immune suppression, cellular plasticity and resistance to radiotherapy and temozolomide. The gene mutations most responsible for the MES-GBM phenotype and particularly *NF1*, are regulators of the ERK MAPK pathway, potentially rendering the tumor susceptible to RASi even in the absence of direct RAS mutations. This susceptibility has been demonstrated with drugs exhibiting off-target RASi effects (e.g., fluvastatin) and with downstream ERK MAPK pathway inhibitors such as MEKi (trametinib). RAS(ON) multi-selective inhibitors therefore represent a conceptually important logical next step that warrants further testing. Daraxonrasib has broad activity against RAS-driven cancers, and a favorable clinical tolerability profile. It is mechanistically suited for *NF1*-mutant MES-GBM in which RAS is constitutively active despite absence of RAS mutation. Beyond direct tumor cell effects, RAS(ON) inhibition holds potential to remodel the immunosuppressive GBM microenvironment toward an anti-tumor state which is a potential therapeutic benefit that distinguishes this approach from conventional chemotherapy.

Dedicated preclinical studies are therefore essential before daraxonrasib can be advanced as a therapeutic candidate for GBM. Priority studies should include orthotopic NF1-deficient and MES-GBM models, rigorous pharmacokinetic and pharmacodynamic assessment of blood–brain and blood–tumor barrier penetration, confirmation of intratumoral target engagement, evaluation of effects on tumor growth and survival, and systematic characterization of microenvironmental remodeling. In parallel, rational combination strategies with radiotherapy, temozolomide, BBB-modulating approaches, or inhibitors of compensatory pathways such as PI3K/AKT/mTOR should be investigated. Collectively, these studies will determine whether RAS(ON) inhibition can translate the molecular vulnerability created by NF1 loss into a viable therapeutic strategy for MES-GBM.

## References

[B1] Ahmed M. Canney M. Carpentier A. Thanou M. Idbaih A. (2023). Unveiling the enigma of the blood-brain barrier in glioblastoma: Current advances from preclinical and clinical studies. *Curr. Opin. Oncol.* 35 522–528. 10.1097/CCO.0000000000000990 37681417 PMC10566587

[B2] Albert L. Karsy M. Murali R. Jhanwar-Uniyal M. (2009). Inhibition of mTOR activates the MAPK pathway in glioblastoma multiforme. *Cancer Genomics Proteomics* 6 255–261. 19996130

[B3] Anastacio Da Costa Carvalho L. Tovbis Shifrin N. Phadke M. S. Emmons M. F. Pechuan-Jorge X. Mbuga F. et al. (2026). RAS(ON) multiselective inhibition drives antitumor immunity in preclinical models of NRAS-mutant melanoma. *Cancer Immunol. Res.* 14 90–106. 10.1158/2326-6066.CIR-25-0744 41186497 PMC12666865

[B4] Anastasaki C. Orozco P. Gutmann D. H. (2022). RAS and beyond: The many faces of the neurofibromatosis type 1 protein. *Dis. Model. Mech.* 15:dmm049362. 10.1242/dmm.049362 35188187 PMC8891636

[B5] Andersen R. Anand A. Harwood D. Kristensen B. (2021). Tumor-associated microglia and macrophages in the glioblastoma microenvironment and their implications for therapy. *Cancers* 13:4255. 10.3390/cancers13174255 34503065 PMC8428223

[B6] Behnan J. Finocchiaro G. Hanna G. (2019). The landscape of the mesenchymal signature in brain tumours. *Brain* 142 847–866. 10.1093/brain/awz044 30946477 PMC6485274

[B7] Berger T. Wen P. Lang-Orsini M. Chukwueke U. (2022). World health organization 2021 classification of central nervous system tumors and implications for therapy for adult-type gliomas: A review. *JAMA Oncol.* 8 1493–1501. 10.1001/jamaoncol.2022.2844 36006639

[B8] Bowman R. Klemm F. Akkari L. Pyonteck S. Sevenich L. Quail D. et al. (2016). Macrophage ontogeny underlies differences in tumor-specific education in brain malignancies. *Cell. Rep.* 17 2445–2459. 10.1016/j.celrep.2016.10.052 27840052 PMC5450644

[B9] Carpentier A. Stupp R. Sonabend A. Dufour H. Chinot O. Mathon B. et al. (2024). Repeated blood-brain barrier opening with a nine-emitter implantable ultrasound device in combination with carboplatin in recurrent glioblastoma: A phase I/II clinical trial. *Nat. Commun.* 15:1650. 10.1038/s41467-024-45818-7 38396134 PMC10891097

[B10] Carracedo A. Ma L. Teruya-Feldstein J. Rojo F. Salmena L. Alimonti A. et al. (2008). Inhibition of mTORC1 leads to MAPK pathway activation through a PI3K-dependent feedback loop in human cancer. *J. Clin. Invest.* 118 3065–3074. 10.1172/JCI34739 18725988 PMC2518073

[B11] Chang M. Sherief M. Ioannou M. Chinnasamy V. Chen L. Frost M. et al. (2024). NF1 expression profiling in IDH-wildtype glioblastoma: Genomic associations and survival outcomes. *Acta Neuropathol. Commun.* 12:172. 10.1186/s40478-024-01875-z 39472976 PMC11520828

[B12] CreggJ. EdwardsA. ChangS. LeeB. KnoxJ. TomlinsonA.et al. (2025). Discovery of daraxonrasib (RMC-6236), a potent and orally bioavailable RAS(ON) multi-selective, noncovalent tri-complex inhibitor for the treatment of patients with multiple RAS-addicted cancers. *J. Med. Chem.* 68 6064–6083. 10.1021/acs.jmedchem.4c02314 40056080

[B13] Davis M. (2016). Glioblastoma: Overview of disease and treatment. *Clin. J. Oncol. Nurs.* 20 S2–S8. 10.1188/16.CJON.S1.2-8 27668386 PMC5123811

[B14] de Gooijer M. de Vries N. Buckle T. Buil L. Beijnen J. Boogerd W. et al. (2018). Improved brain penetration and antitumor efficacy of temozolomide by inhibition of ABCB1 and ABCG2. *Neoplasia* 20 710–720. 10.1016/j.neo.2018.05.001 29852323 PMC6030392

[B15] Digiovanni S. Lorenzati M. Bianciotto O. Godel M. Fontana S. Akman M. et al. (2024). Blood-brain barrier permeability increases with the differentiation of glioblastoma cells in vitro. *Fluids Barriers CNS* 21:89. 10.1186/s12987-024-00590-0 39487455 PMC11529439

[B16] Duan M. Cao R. Yang Y. Chen X. Liu L. Ren B. et al. (2024). Blood-brain barrier conquest in glioblastoma nanomedicine: Strategies, clinical advances, and emerging challenges. *Cancers* 16:3300. 10.3390/cancers16193300 39409919 PMC11475686

[B17] Ebright R. Dilly J. Shaw A. Aguirre A. (2025). Response and resistance to RAS inhibition in cancer. *Cancer Discov*. 15 1325–1349. 10.1158/2159-8290.CD-25-0349 40293709 PMC12226231

[B18] Fisher J. Adamson D. (2021). Current FDA-approved therapies for high-grade malignant gliomas. *Biomedicines* 9:324. 10.3390/biomedicines9030324 33810154 PMC8004675

[B19] Gabrusiewicz K. Li X. Wei J. Hashimoto Y. Marisetty A. Ott M. et al. (2018). Glioblastoma stem cell-derived exosomes induce M2 macrophages and PD-L1 expression on human monocytes. *Oncoimmunology* 7:e1412909. 10.1080/2162402X.2017.1412909 29632728 PMC5889290

[B20] Gerstner E. Emblem K. Chang K. Vakulenko-Lagun B. Yen Y. Beers A. et al. (2020). Bevacizumab reduces permeability and concurrent temozolomide delivery in a subset of patients with recurrent glioblastoma. *Clin. Cancer Res.* 26 206–212. 10.1158/1078-0432.CCR-19-1739 31558474 PMC7139851

[B21] Ghiaseddin A. Peters K. (2015). Use of bevacizumab in recurrent glioblastoma. *CNS Oncol.* 4 157–169. 10.2217/cns.15.8 25906439 PMC6088330

[B22] Guo X. Pan Y. Gutmann D. (2019). Genetic and genomic alterations differentially dictate low-grade glioma growth through cancer stem cell-specific chemokine recruitment of T cells and microglia. *Neuro Oncol.* 21 1250–1262. 10.1093/neuonc/noz080 31111915 PMC6784288

[B23] Habibi M. Mirjani M. Ahmadvand M. Delbari P. Alasti O. (2024). The safety and efficacy of dabrafenib and trametinib in patients with glioma: A systematic review and meta-analysis. *Eur. J. Clin. Pharmacol.* 80 639–656. 10.1007/s00228-024-03635-3 38345637

[B24] Han C. Jeong M. Jang S. (2017). Structure, signaling and the drug discovery of the Ras oncogene protein. *BMB Rep.* 50 355–360. 10.5483/bmbrep.2017.50.7.062 28571593 PMC5584742

[B25] Holderfield M. Lee B. Jiang J. Tomlinson A. Seamon K. Mira A. et al. (2024). Concurrent inhibition of oncogenic and wild-type RAS-GTP for cancer therapy. *Nature* 629 919–926. 10.1038/s41586-024-07205-6 38589574 PMC11111408

[B26] Idbaih A. Canney M. Belin L. Desseaux C. Vignot A. Bouchoux G. et al. (2019). Safety and feasibility of repeated and transient blood-brain barrier disruption by pulsed ultrasound in patients with recurrent glioblastoma. *Clin. Cancer Res.* 25 3793–3801. 10.1158/1078-0432.CCR-18-3643 30890548

[B27] Iturrioz-Rodríguez N. Piccardi F. Bertorelli R. Ciofani G. (2024). Establishment of an orthotopic glioblastoma mouse model for preclinical studies. *Methods Cell Biol.* 185 49–65. 10.1016/bs.mcb.2024.02.004 38556451

[B28] Jeon H. Wang S. Song J. Gill H. Cheng H. (2025). Update 2025: Management of non-small-cell *lung* cancer. *Lung* 203:53. 10.1007/s00408-025-00801-x 40133478 PMC11937135

[B29] Jiang J. Jiang L. Maldonato B. Wang Y. Holderfield M. Aronchik I. et al. (2024). Translational and therapeutic evaluation of RAS-GTP inhibition by RMC-6236 in RAS-driven cancers. *Cancer Discov.* 14 994–1017. 10.1158/2159-8290.CD-24-0027 38593348 PMC11149917

[B30] Krauß D. Moreno-Viedma V. Adachi-Fernandez E. de Sá Fernandes C. Genger J. Fari O. et al. (2025). EGFR controls transcriptional and metabolic rewiring in KRASG12D colorectal cancer. *EMBO Mol. Med.* 17 1355–1392. 10.1038/s44321-025-00240-4 40329096 PMC12162862

[B31] Kumari S. Ahsan S. Kumar J. Kondapi A. Rao N. (2017). Overcoming blood brain barrier with a dual purpose Temozolomide loaded Lactoferrin nanoparticles for combating glioma (SERP-17-12433). *Sci. Rep.* 7:6602. 10.1038/s41598-017-06888-4 28747713 PMC5529576

[B32] Lee J. K. Sivakumar S. Schrock A. B. Madison R. Fabrizio D. Gjoerup O. et al. (2022). Comprehensive pan-cancer genomic landscape of KRAS altered cancers and real-world outcomes in solid tumors. *NPJ Precis. Oncol.* 6:91. 10.1038/s41698-022-00334-z 36494601 PMC9734185

[B33] Lee Y. Tan Y. Oon C. (2018). Molecular targeted therapy: Treating cancer with specificity. *Eur. J. Pharmacol.* 834 188–196. 10.1016/j.ejphar.2018.07.034 30031797

[B34] Levy A. Bryant J. Matichak D. Onishi S. Banasavadi-Siddegowda Y. K. (2025). MEK inhibition in glioblastoma: Current perspectives and future directions. *Int. J. Mol. Sci.* 26:6875. 10.3390/ijms26146875 40725122 PMC12295435

[B35] Liang Y. Zhi S. Qiao Z. Meng F. (2023). Predicting blood-brain barrier permeation of erlotinib and JCN037 by molecular simulation. *J. Membr. Biol.* 256 147–157. 10.1007/s00232-022-00274-6 36441253

[B36] Lo H. (2010). Targeting Ras-RAF-ERK and its interactive pathways as a novel therapy for malignant gliomas. *Curr. Cancer Drug Targets* 10 840–848. 10.2174/156800910793357970 20718706 PMC3615246

[B37] Long C. Yuan L. Wei W. Li J. (2022). Overcoming chemoresistance in glioblastoma by fluvastatin via prenylation-dependent inhibition of Ras signaling. *Hum. Exp. Toxicol.* 41:9603271221125934. 10.1177/09603271221125934 36171180

[B38] Ma R. Black A. Qian B. (2022). Macrophage diversity in cancer revisited in the era of single-cell omics. *Trends Immunol.* 43 546–563. 10.1016/j.it.2022.04.008 35690521

[B39] Marei H. Althani A. Afifi N. Hasan A. Caceci T. Pozzoli G. et al. (2021). p53 signaling in cancer progression and therapy. *Cancer Cell. Int.* 21:703. 10.1186/s12935-021-02396-8 34952583 PMC8709944

[B40] Marques C. Unterkircher T. Kroon P. Oldrini B. Izzo A. Dramaretska Y. et al. (2021). NF1 regulates mesenchymal glioblastoma plasticity and aggressiveness through the AP-1 transcription factor FOSL1. *Elife* 10:e64846. 10.7554/eLife.64846 34399888 PMC8370767

[B41] Mesfin F. B. Karsonovich T. Al-Dhahir M. A. (2024). “Gliomas,” in *StatPearls*, (Treasure Island, FL: StatPearls Publishing). 28722904

[B42] Minichmayr I. Knaack U. Gojo J. Senfter D. Haberler C. Azizi A. et al. (2024). Distribution of bevacizumab into the cerebrospinal fluid of children and adolescents with recurrent brain tumors. *Paediatr. Drugs* 26 429–440. 10.1007/s40272-024-00624-y 38587585 PMC11192692

[B43] Mirzaei R. McNeil R. D’Mello C. Wong B. Sarkar S. Visser F. et al. (2026). Spatial single-cell profiling identifies protein kinase Cδ-expressing microglia with anti-tumor function in glioblastoma. *iScience* 29:114281. 10.1016/j.isci.2025.114281 41503214 PMC12768871

[B44] Mizukami T. Izawa N. Nakajima T. Sunakawa Y. (2019). Targeting EGFR and RAS/RAF signaling in the treatment of metastatic colorectal cancer: From current treatment strategies to future perspectives. *Drugs* 79 633–645. 10.1007/s40265-019-01113-0 30968289

[B45] Molina J. Adjei A. A. (2006). The Ras/Raf/MAPK pathway. *J. Thoracic Oncol.* 1 7–9. 17409820

[B46] Moore A. Rosenberg S. McCormick F. Malek S. (2020). RAS-targeted therapies: Is the undruggable drugged? *Nat. Rev. Drug Discov.* 19 533–552. 10.1038/s41573-020-0068-6 32528145 PMC7809886

[B47] Nokin M. Mira A. Patrucco E. Ricciuti B. Cousin S. Soubeyran I. et al. (2024). RAS-ON inhibition overcomes clinical resistance to KRAS G12C-OFF covalent blockade. *Nat. Commun.* 15:7554. 10.1038/s41467-024-51828-2 39215000 PMC11364849

[B48] Ochocka N. Segit P. Walentynowicz K. Wojnicki K. Cyranowski S. Swatler J. et al. (2021). Single-cell RNA sequencing reveals functional heterogeneity of glioma-associated brain macrophages. *Nat. Commun.* 12:1151. 10.1038/s41467-021-21407-w 33608526 PMC7895824

[B49] Oprita A. Baloi S. Staicu G. Alexandru O. Tache D. Danoiu S. et al. (2021). Updated insights on EGFR signaling pathways in glioma. *Int. J. Mol. Sci.* 22 587. 10.3390/ijms22020587 33435537 PMC7827907

[B50] Ortiz R. Perazzoli G. Cabeza L. Jiménez-Luna C. Luque R. Prados J. et al. (2021). Temozolomide: An updated overview of resistance mechanisms, nanotechnology advances and clinical applications. *Curr. Neuropharmacol.* 19 513–537. 10.2174/1570159X18666200626204005 32589560 PMC8206461

[B51] Perurena N. Cichowski K. (2024). Finding the sweet spot: Targeting RAS in tumors while sparing normal tissue. *Cancer Cell.* 42 943–945. 10.1016/j.ccell.2024.05.020 38861932

[B52] Philpott C. Tovell H. Frayling I. Cooper D. Upadhyaya M. (2017). The NF1 somatic mutational landscape in sporadic human cancers. *Hum. Genomics* 11:13. 10.1186/s40246-017-0109-3 28637487 PMC5480124

[B53] Prior I. Hood F. Hartley J. (2020). The frequency of ras mutations in cancer. *Cancer Res.* 80 2969–2974. 10.1158/0008-5472.CAN-19-3682 32209560 PMC7367715

[B54] Punekar S. Velcheti V. Neel B. Wong K. (2022). The current state of the art and future trends in RAS-targeted cancer therapies. *Nat. Rev. Clin. Oncol.* 19 637–655. 10.1038/s41571-022-00671-9 36028717 PMC9412785

[B55] Rao R. Han R. Ogurek S. Xue C. Wu L. Zhang L. et al. (2022). Glioblastoma genetic drivers dictate the function of tumor-associated macrophages/microglia and responses to CSF1R inhibition. *Neuro Oncol.* 24 584–597. 10.1093/neuonc/noab228 34562087 PMC8972285

[B56] Sarkaria J. Hu L. Parney I. Pafundi D. Brinkmann D. Laack N. et al. (2018). Is the blood-brain barrier really disrupted in all glioblastomas? A critical assessment of existing clinical data. *Neuro Oncol.* 20 184–191. 10.1093/neuonc/nox175 29016900 PMC5777482

[B57] Schreck K. Allen A. Wang J. Pratilas C. (2020). Combination MEK and mTOR inhibitor therapy is active in models of glioblastoma. *Neurooncol. Adv.* 2:vdaa138. 10.1093/noajnl/vdaa138 33235998 PMC7668446

[B58] Sharma P. Aaroe A. Liang J. Puduvalli V. (2023). Tumor microenvironment in glioblastoma: Current and emerging concepts. *Neurooncol. Adv.* 5:vdad009. 10.1093/noajnl/vdad009 36968288 PMC10034917

[B59] Shen Y. Thng D. Wong A. Toh T. (2024). Mechanistic insights and the clinical prospects of targeted therapies for glioblastoma: A comprehensive review. *Exp. Hematol. Oncol.* 13:40. 10.1186/s40164-024-00512-8 38615034 PMC11015656

[B60] Solomou G. Young A. Bulstrode H. (2024). Microglia and macrophages in glioblastoma: Landscapes and treatment directions. *Mol. Oncol.* 18 2906–2926. 10.1002/1878-0261.13657 38712663 PMC11619806

[B61] Song Y. Bi Z. Liu Y. Qin F. Wei Y. Wei X. (2023). Targeting RAS-RAF-MEK-ERK signaling pathway in human cancer: Current status in clinical trials. *Genes Dis.* 10 76–88. 10.1016/j.gendis.2022.05.006 37013062 PMC10066287

[B62] Spira A. I. Starodub A. Arbour K. Sommerhalder C. Wolpin B. et al. (2023). Abstract PR010: Preliminary safety and pharmacokinetic profiles of RMC-6236, a first-in-class, RAS-selective, tri-complex RAS^MULTI^ (ON) inhibitor in patients with KRAS-mutant solid tumors on the Phase 1 trial RMC-6236-001. *Mol. Cancer Ther.* 22:PR010. 10.1158/1535-7163.TARG-23-PR010

[B63] Stupp R. Mason W. van den Bent M. Weller M. Fisher B. Taphoorn M. et al. (2005). Radiotherapy plus concomitant and adjuvant temozolomide for glioblastoma. *N. Engl. J. Med.* 352 987–996. 10.1056/NEJMoa043330 15758009

[B64] Terstappen G. Meyer A. Bell R. Zhang W. (2021). Strategies for delivering therapeutics across the blood-brain barrier. *Nat. Rev. Drug Discov.* 20 362–383. 10.1038/s41573-021-00139-y 33649582

[B65] Tripathy D. Panda L. Biswal S. Barhwal K. (2024). Insights into the glioblastoma tumor microenvironment: Current and emerging therapeutic approaches. *Front. Pharmacol.* 15:1355242. 10.3389/fphar.2024.1355242 38523646 PMC10957596

[B66] Vadla R. Miki S. Taylor B. Kawauchi D. Jones B. Nathwani N. et al. (2023). Glioblastoma mesenchymal transition and invasion are dependent on a NF-kappaB/BRD2 chromatin complex. *bioRxiv [Preprint]* 10.1101/2023.07.03.546613 37461511 PMC10349949

[B67] van den Bent M. Brandes A. Rampling R. Kouwenhoven M. Kros J. Carpentier A. et al. (2009). Randomized phase II trial of erlotinib versus temozolomide or carmustine in recurrent glioblastoma: EORTC brain tumor group study 26034. *J. Clin. Oncol.* 27 1268–1274. 10.1200/JCO.2008.17.5984 19204207 PMC2667826

[B68] van den Bent M. Klein M. Smits M. Reijneveld J. French P. Clement P. et al. (2018). Bevacizumab and temozolomide in patients with first recurrence of WHO grade II and III glioma, without 1p/19q co-deletion (TAVAREC): A randomised controlled phase 2 EORTC trial. *Lancet Oncol.* 19 1170–1179. 10.1016/S1470-2045(18)30362-0 30115593

[B69] Wang Q. Hu B. Hu X. Kim H. Squatrito M. Scarpace L. et al. (2018). Tumor evolution of glioma-intrinsic gene expression subtypes associates with immunological changes in the microenvironment. *Cancer Cell.* 33:152. 10.1016/j.ccell.2017.12.012 29316430 PMC5892424

[B70] Wang Q. Hu B. Hu X. Kim H. Squatrito M. Scarpace L. et al. (2017). Tumor evolution of glioma-intrinsic gene expression subtypes associates with immunological changes in the microenvironment. *Cancer Cell*. 32 42–56.e6. 10.1016/j.ccell.2017.06.003 28697342 PMC5599156

[B71] Wang S. Wang J. Chen Z. Luo J. Guo W. Sun L. et al. (2024). Targeting M2-like tumor-associated macrophages is a potential therapeutic approach to overcome antitumor drug resistance. *NPJ Precis. Oncol.* 8:31. 10.1038/s41698-024-00522-z 38341519 PMC10858952

[B72] Wang Y. Tian J. Liu D. Li T. Mao Y. Zhu C. (2024). Microglia in radiation-induced brain injury: Cellular and molecular mechanisms and therapeutic potential. *CNS Neurosci. Ther.* 30:e14794. 10.1111/cns.14794 38867379 PMC11168970

[B73] Wei S. Chang L. Zhong Y. (2024). The efficacy and adverse events of bevacizumab combined with temozolomide in the treatment of glioma: A systemic review and meta-analysis of randomized controlled trials. *Front. Med.* 11:1419038. 10.3389/fmed.2024.1419038 39015784 PMC11250252

[B74] Wu D. Chen Q. Chen X. Han F. Chen Z. Wang Y. (2023). The blood-brain barrier: Structure, regulation, and drug delivery. *Signal. Transduct Target. Ther.* 8:217. 10.1038/s41392-023-01481-w 37231000 PMC10212980

[B75] Xu H. Zong H. Ma C. Ming X. Shang M. Li K. et al. (2017). Epidermal growth factor receptor in glioblastoma. *Oncol. Lett.* 14 512–516. 10.3892/ol.2017.6221 28693199 PMC5494611

[B76] Xu W. Zhang T. Zhou Y. Mao Y. (2024). Fluvastatin prevents lung metastasis in triple-negative breast cancer by triggering autophagy via the RhoB/PI3K/mTOR pathway. *Exp. Cell. Res.* 435:113893. 10.1016/j.yexcr.2023.113893 38123008

[B77] Yaghoubi F. Ebrahimi S. Gorji A. Khaleghi Ghadiri M. (2026). Stereotactic intracranial implantation of patient-derived glioblastoma cells in rats: A xenograft modeling approach. *J. Neurosci. Methods* 427:110658. 10.1016/j.jneumeth.2025.110658 41391715

[B78] Yalamarty S. Filipczak N. Li X. Subhan M. Parveen F. Ataide J. et al. (2023). Mechanisms of resistance and current treatment options for glioblastoma multiforme (GBM). *Cancers* 15:2116. 10.3390/cancers15072116 37046777 PMC10093719

[B79] Yu K. Lin C. Hatcher A. Lozzi B. Kong K. Huang-Hobbs E. et al. (2020). PIK3CA variants selectively initiate brain hyperactivity during gliomagenesis. *Nature* 578 166–171. 10.1038/s41586-020-1952-2 31996845 PMC7577741

[B80] Zhang Z. Chen J. Huo X. Zong G. Huang K. Cheng M. et al. (2021). Identification of a mesenchymal-related signature associated with clinical prognosis in glioma. *Aging* 13 12431–12455. 10.18632/aging.202886 33875619 PMC8148476

